# Application value of oral targeted drug management program led by specialist nurses in continuous nursing for lung cancer patients

**DOI:** 10.3389/fmed.2025.1693815

**Published:** 2025-11-19

**Authors:** Xinyu Zhang, Tingting Lian

**Affiliations:** Department of Respiratory and Critical Care Medicine, The First Affiliated Hospital of Suzhou University, Suzhou, Jiangsu, China

**Keywords:** non-small cell lung cancer, oral targeted drug therapy, specialist nurses, medication compliance, continuity of care

## Abstract

**Objective:**

Our research attempted to clarify impact of oral targeted drug management program led by specialist nurses on prognosis of non-small cell lung cancer (NSCLC) patients under treatment with oral targeted drugs out of hospital.

**Methods:**

NSCLC patients visiting our hospital enrollment as research cohort. The 80 patients received enrollment in our research and division into intervention group and control group through random number table method. The control group underwent conventional nursing. Based on control group, intervention group underwent an oral targeted drug management program led by specialist nurses. Patients received follow-up at 2, 4 and 6 months after discharge.

**Results:**

At follow-up of 4 and 6 months, incidence of missed medication events in intervention group displayed depletion relative to that in controls. At follow-up of 4 and 6 months, patients’ medication compliance in intervention group displayed elevation relative to that in controls. Incidence of adverse drug reactions displayed depletion in intervention group. At follow-up of 2, 4 and 6 months, incidence of erythra in intervention group displayed depletion relative to that in controls. At follow-up of 6 months, general health, physical functioning and social functioning scores in intervention group displayed elevation relative to those in controls. The scores of above indicators in both groups at follow-up of 6 months displayed elevation relative to those at follow-up of 2 months.

**Conclusion:**

An oral targeted drug management program led by specialist nurses can effectively elevate patients’ compliance, reduce incidence of adverse drug reactions and improve patients’ quality of life.

## 1 Introduction

Targeted drug therapy is one of the new approaches to treating tumors in recent years, which is widely used in non-small cell lung cancer (NSCLC) due to its characteristics of targeting, safety, and convenience (1–3). Several scholars have illustrated that for advanced NSCLC patients who cannot accept or tolerate chemotherapy, oral targeted drug therapy can be taken out of hospital, which can reduce tumor recurrence and metastasis, elevate patients’ survival, and improve patients’ quality of life (4–6). Nevertheless, during period of out-of-hospital therapy for NSCLC patients, due to lack of effective medical supervision, patients’ medication compliance and treatment effectiveness are affected to some extent, and due to patients’ limited ability to handle drug toxicity and side effects, these may lead to occurrence of adverse prognosis (7). Thus, it is of great significance to develop a new drug management plan for NSCLC patients undergoing oral targeted drug therapy out of hospital, effectively monitor their regular medication administration, and improve their ability to handle adverse drug reactions. Our research attempted to clarify impact of oral targeted drug management program led by specialist nurses on prognosis of NSCLC patients under treatment with oral targeted drugs out of hospital. The report is as follows.

## 2 Materials and methods

### 2.1 General data

NSCLC patients visiting our hospital from December 2021 to November 2022 received enrollment as research cohort. Inclusion criteria: (1) All patients were pathologically diagnosed as NSCLC, and international lung cancer primary tumor-node-metastasis (TNM) staging (8) was stage IIIB or IV; (2) had not received surgery or chemotherapy; (3) positive in epidermal growth factor receptor (EGFR), and physical activity status (9) scored 1–2 points; (4) estimated survival period was > 6 months, and patients underwent treatment with oral EGFR tyrosine kinase inhibitor icotinib out of hospital; (5) informed consent and voluntary participation in research. Exclusion criteria: (1) With severe cardiovascular and cerebrovascular diseases; (2) with cognitive impairment, mental illness, etc., and unable to cooperate with therapy; (3) pregnant or lactating women; (4) missing visits or incomplete information. Finally, 80 patients received enrollment in our research and division into intervention group (40 cases) and control group (40 cases) through random number table method. This research received reviewing and approval by the Medical Ethics Committee of our hospital.

### 2.2 Methods

The control group underwent conventional nursing. During the hospitalization period, the nursing staff provided the patients with education on diseases-related knowledge as well as oral guidance on targeted drug treatment, including specific dosage information for the icotinib targeted drug. The initial recommended dose of icotinib is 125 mg (Betta Pharmaceuticals Co., Ltd., Zhejiang, China) three times a day, taken orally. Each cycle lasted for 3 weeks, and the entire treatment process consisted of 4 cycles. To ensure the safety of patients’ medication use and to promptly monitor any possible adverse reactions in the blood system during the treatment with icotinib, patients were required to undergo blood tests. Before starting the treatment with icotinib, the patient needed to undergo a comprehensive blood test, including blood routine, liver function, kidney function and coagulation function, to establish baseline data and provide reference for subsequent treatment and monitoring. Within the first week after the start of treatment, a blood routine test was conducted once a day to closely observe the impact of the drug on bone marrow hematopoietic function and promptly detect possible adverse reactions such as leukopenia and thrombocytopenia. At the same time, liver function and kidney function tests were conducted every 3 days to assess the impact of the drug on liver and kidney function. Starting from the second week of treatment, if the blood routine, liver function and kidney function indicators were stable and no serious adverse reactions occurred, the frequency of blood routine tests was adjusted to once every 3 days, and the frequency of liver function and kidney function tests was adjusted to once a week. At the end of each 3-week treatment cycle, a comprehensive blood test was conducted, including blood routine, liver function, kidney function and coagulation function, to evaluate the overall condition of the patient and the drug tolerance during this cycle. If the patient experienced any discomfort symptoms during the medication process, such as fever, fatigue, bleeding tendency, or abnormal fluctuations in test indicators, such as significantly decreased white blood cell count, significantly elevated transaminase, the test frequency was increased immediately. In some cases, blood routine tests were conducted daily, and liver function and kidney function tests were conducted every 1–2 days, and corresponding treatments were carried out according to the specific situation. The nursing staff explained in detail to the patients and their families the importance of the drug dosage, the administration method, and the possible situations resulting from dose adjustment, so that the patients, their families, and the nursing staff can understand the relevant knowledge of the disease and treatment, and thus cooperate actively with the treatment and nursing work. Most patients who received icotinib targeted therapy were at an advanced stage, and they experienced certain psychological symptoms such as tension, anxiety, worry and fear, and were unfamiliar with the targeted treatment. At this point, nursing staff provided psychological guidance for patients, communicated with them more, provided comfort, relieved their ideological concerns, and allowed them to receive therapy at ease; nursing staff explained efficacy and price of targeted drugs to patients and their families, and informed patients of possible adverse reactions and simple treatment methods during treatment process; common adverse reactions include erythra, diarrhea and nausea. For different symptoms, the treatment methods are as follows: For erythra, if the symptoms are mild, it is recommended to use mild moisturizing products to keep the skin moist and avoid using highly irritating cosmetics or skin care products; if the erythra are severe and accompanied by itching and pain, it is necessary to inform the doctor immediately and follow the doctor’s advice to use corresponding topical medications for treatment. For diarrhea, if the number of daily diarrhea episodes is low and the changes in stool consistency are not obvious, it is recommended to guide the patient to adjust their diet, mainly consuming light and easily digestible foods, avoiding eating raw, greasy, spicy and other irritating foods, and at the same time, pay attention to replenishing water and electrolytes; if the diarrhea occurs frequently, accompanied by abdominal pain, fever and other symptoms, it is necessary to seek medical attention immediately and undergo further examinations and treatments. For nausea, it is recommended that patients consume some light snacks such as biscuits before and after taking medication to relieve the nausea symptoms; if the nausea symptoms are severe and affect eating and normal life, it is necessary to contact the doctor promptly and consider adjusting the drug dosage or changing the treatment plan. Before discharge, patients were given guidance on method and timing of targeted drug use, emphasized importance of taking drugs on time and in an appropriate amount. Nursing staff advised patients to record their symptoms and duration in detail if they experienced adverse reactions during medication; if adverse reactions were severe, patients promptly contacted a doctor or visited a local hospital.

Intervention group: Based on control group, intervention group underwent an oral targeted drug management program led by specialist nurses. (1) Establish an intervention team: The intervention team consists of 4 specialized nurses from the Department of Respiratory Oncology. All team members have obtained qualification of tumor specialist nurse and qualification certificate of knowledge training on quality management practices in drug clinical trials and have been engaged in tumor specialist nursing for over 5 years. They have participated in out-of-hospital continuous nursing work, and have strong tumor specialist nursing skills, management skills and communication skills. Team members have received unified training to master purpose, significance, etc., of this research. (2) Filing: Establish patients’ out-of-hospital treatment file, including patients’ follow-up plan, medication status, adverse drug reactions, nursing interventions, follow-up frequency, etc. (3) Medication execution record card: When patients are discharged from hospital, medication execution record cards are issued, allowing them to record on card each time they take medication after discharge. (4) Registration form for adverse drug reactions: The patients are issued with a registration form for adverse drug reactions upon discharge. If patients experience adverse reactions during medication, they should promptly record status on registration form and feed back to intervention team members for symptomatic treatment. Adverse reactions of targeted drugs were recorded in accordance with the 2009 HHS/NIH/NCI Common Adverse Reaction Event Evaluation Standard. (5) Care and reexamination: Team members should send health medication guidance and daily humanistic care to patients every week through mobile phone SMS and send reminders of time of return to hospital for reexamination according to follow-up time. (6) Follow up: Team members should conduct a weekly telephone follow-up to monitor and understand patients’ condition and medication use. If symptoms worsen, team members should instruct patients to return to hospital and report to doctors in a timely manner. Team members should understand patients’ psychological and living conditions, and timely adjust nursing interventions based on each person’s situation. Team members should communicate with patients’ family or primary caregivers to understand patients’ therapy status and needs. (7) Social support: Team members should establish a WeChat group of patients and medical personnel for therapy out of hospital. Responsible nurses and patients should communicate through WeChat, supervise and manage each other, and establish a people-oriented, harmonious, and harmonious doctor-patient relationship. Social support should be elevated.

### 2.3 Observation indicators

Patients received follow-up at 2, 4 and 6 months after discharge. The occurrence of missed medication events, adverse drug reactions, medication compliance and patients’ quality of life received recording. (1) Occurrence of missed medication events: The situation received registration according to patients’ medication execution record card and quantity of drugs received counting to determine missed medication. (2) Occurrence of adverse drug reactions: Statistics shall be made based on patients’ results during their return to hospital for reexamination or records in adverse drug reaction registration form. Adverse drug reactions include elevated transaminases, erythra, diarrhea and agranulocytosis. (3) Evaluation of medication compliance: Medication compliance received evaluation through Drug Compliance Scale. The full scores are 8 points, with a score of <6 points indicating low compliance, 6–7 points indicating moderate compliance, and 8 points indicating high compliance. (4) Quality of life: Quality of life received evaluation through European Cancer Research and Organization Core Quality of Life Scale (10) + lung cancer specific sections. General health and physical, role, cognitive, emotional, and social functioning received evaluation.

### 2.4 Statistical analysis

SPSS 22.0 software received utilization for statistical analysis. The measurement data received expression in (x ± s) and comparison through *t*-test. Repeated measurement data received comparison through analysis of variance. The counting data received description by number of cases (percentage) and comparison through *χ*^2^. The difference was statistically significant with *p* < 0.05.

## 3 Results

### 3.1 Comparison of general data between both groups

Intervention group: Age: (53.55 ± 6.68) years; 15 males and 25 females; TNM staging: stage III-B in 7 cases and stage IV in 33 cases. Control group: Age: (55.18 ± 5.15) years; 11 males and 29 females; TNM staging: stage III-B in 5 cases and stage IV in 35 cases. The age, gender, and TNM staging displayed no difference between both groups (*p* > 0.05; Table 1), which is comparable.

**Table 1:** General data in both groups.

Groups	*N*	Age	Gender		TNM	
			Male	Female	III B	IV
Control group	40	55.18 ± 5.15	11	29	5	35
Intervention group	40	54.55 ± 5.68	15	25	7	33
*χ*^2^/*t*		0.964	0.912		0.392	
*p*		0.341	0.34		0.531	

### 3.2 Comparison of incidence of missed medication events between both groups

At follow-up of 4 and 6 months, incidence of missed medication events in intervention group displayed depletion relative to that in controls (*p* < 0.05; Table 2).

**Table 2:** Missed medication events in both groups.

Groups	*N*	Follow up for 2 months	Follow up for 4 months	Follow up for 6 months
Control group	40	2	7	9
Intervention group	40	1	1	2
*χ* ^2^		0.346	5	5.165
*p*		0.556	0.025	0.023

### 3.3 Comparison of medication compliance between both groups

At follow-up of 4 and 6 months, patients’ medication compliance in intervention group displayed elevation relative to that in controls (*p* < 0.05; Table 3).

**Table 3:** Medication compliance in both groups.

Groups	*N*	Follow up for 2 months			Follow up for 4 months			Follow up for 4 months		
		Low compliance	Moderate compliance	High compliance	Low compliance	Moderate compliance	High compliance	Low compliance	Moderate compliance	High compliance
Control group	40	0	6	34	4	8	28	9	10	21
Intervention group	40	0	4	36	0	3	37	0	4	36
*χ* ^2^		0.457			7.519			15.519		
*p*		0.499			0.023			<0.001		

### 3.4 Comparison of incidence of adverse drug reactions between both groups

Our study analyzed the incidence of elevated transaminases, erythra, diarrhea and agranulocytosis in two groups. At follow-up of 2 months, the incidence of erythra in intervention group was lower relative to that in controls (*p* < 0.05). At follow-up of 4 months, the incidence of erythra and diarrhea in intervention group was lower relative to that in controls (*p* < 0.05). At follow-up of 6 months, the incidence of erythra in intervention group was lower relative to that in controls (*p* < 0.05; Table 4). During the entire follow-up period of the study, no patients with the aforementioned side effects required hospitalization. The elevated transaminase levels observed in the patients were mostly mild. After adjusting the diet, providing adequate rest, and closely monitoring liver function indicators, the transaminase levels gradually returned to normal. For the erythra, patients with mild symptoms could be relieved through skin care and avoiding irritants, while those with more severe symptoms could be effectively controlled after using topical medications under the guidance of a doctor. For patients with diarrhea, the symptoms improved after adjusting the diet structure, replenishing water and electrolytes. Patients with agranulocytosis were relatively few in number and of mild severity. After close observation and appropriate treatment, no serious complications occurred and no further hospitalization was required for further treatment.

**Table 4:** Adverse drug reactions in both groups.

Groups	*N*	Follow up for 2 months				Follow up for 4 months				Follow up for 6 months			
		Elevated transaminase	Erythra	Diarrhea	Agranulocytosis	Elevated transaminase	Erythra	Diarrhea	Agranulocytosis	Elevated transaminase	Erythra	Diarrhea	Agranulocytosis
Control group	40	10	26	11	5	8	14	10	5	4	12	8	1
Intervention group	40	11	13	10	8	3	6	0	3	5	4	6	0
*χ* ^2^		0.065	8.455	0.065	0.827	2.635	4.267	11.429	0.556	0.125	5	0.346	1.013
*p*		0.799	0.004	0.799	0.363	0.105	0.039	0.001	0.456	0.723	0.025	0.556	0.314

### 3.5 Comparison of quality of life between both groups

At follow-up of 6 months, general health, physical functioning and social functioning scores in intervention group displayed elevation relative to those in controls (*p* < 0.05). The scores of above indicators in both groups at follow-up of 6 months displayed elevation relative to those at follow-up of 2 months (*p* < 0.05; Table 5).

**Table 5:** Quality of life in both groups.

Groups	*N*	Follow up for 2 months					
		General health	Physical functioning	Role functioning	Emotional functioning	Cognitive functioning	Social functioning
Control group	40	49.22 ± 13.72	66.51 ± 10.14	65.12 ± 14.00	62.60 ± 10.97	64.73 ± 18.63	62.02 ± 16.81
Intervention group	40	47.96 ± 16.20	68.44 ± 9.27	64.81 ± 15.99	68.70 ± 13.20	67.41 ± 15.88	70.00 ± 16.14
*t*		0.155	1.015	2.799	1.481	2.793	1.702
*p*		0.877	0.316	0.008	0.147	0.008	0.097

Groups	*N*	Follow up for 4 months					
		General health	Physical functioning	Role functioning	Emotional functioning	Cognitive functioning	Social functioning
Control group	40	66.67 ± 10.30	89.92 ± 7.76	78.29 ± 12.89	78.87 ± 8.61	79.07 ± 10.98	77.91 ± 15.75
Intervention group	40	69.44 ± 9.41	91.70 ± 6.39	81.11 ± 13.59	83.52 ± 7.85	84.07 ± 10.64	84.81 ± 13.22
*t*		0.66	0.954	1.122	1.478	1.116	1.914
*p*		0.513	0.346	0.269	0.147	0.271	0.063

Groups	*N*	Follow up for 6 months					
		General health	Physical functioning	Role functioning	Emotional functioning	Cognitive functioning	Social functioning
Control group	40	66.86 ± 9.72^#^	81.39 ± 7.36^#^	76.74 ± 14.16^#^	81.00 ± 7.79^#^	78.68 ± 12.78^#^	73.64 ± 15.11^#^
Intervention group	40	71.30 ± 10.30^*,#^	87.78 ± 7.03^*,#^	80.00 ± 14.92^#^	80.56 ± 9.74^#^	84.44 ± 13.49^#^	80.00 ± 12.62^*,#^
*t*		4.011	3.818	1.437	0.734	1.966	2.268
*p*		< 0.001	< 0.001	0.159	0.467	0.056	0.029

Versus controls, ^*^p < 0.05; versus 2 months, ^#^*p* < 0.05.

## 4 Discussion

NSCLC is one of the most common malignancies clinically, whose incidence and mortality are elevating year by year, which seriously threatens patients’ life and health (11–13). Targeted therapy, a new type of tumor treatment method that has emerged in recent years, exerts a good therapeutic effect on advanced or chemotherapy-resistant NSCLC patients, and is favored by patients and medical personnel (14, 15). Nevertheless, toxic and side effects caused by long-term targeted drug therapy cannot be ignored (16). Patients with NSCLC who receive oral targeted drug treatment outside the hospital often miss doses of medication and fail to receive timely treatment for adverse reactions, which seriously affects their continuous treatment and therapeutic effect. Thus, figuring out a management plan for patients under oral targeted drug therapy out of hospital is of great significance for effective supervision and service for patients.

The compliance of oral targeted drugs taken out of hospital directly affects patients’ therapeutic effect (17). Research has demonstrated that tumor patients have high compliance when they first undergo oral targeted drug therapy out of hospital; nevertheless, over time, patients have lost effective supervision of medical personnel, incidence of missed medication events has gradually exhibited elevation, and medication compliance has exhibited depletion, affecting treatment effectiveness (18). Our hospital adopted an oral targeted drug management program led by specialist nurses to manage patients and established an intervention team composed of specialist oncology nurses. When patients were discharged from hospital, they were issued with medication execution record cards and adverse drug reaction registration forms. Team members instructed patients to promptly record after each medication, promptly register and provide timely feedback when adverse reactions occurred. Intervention team members communicated with patients through SMS and WeChat to understand their condition and medication status, urged them to take medication on time, and conducted regular follow-up visits to improve their medication compliance. Herein, at follow-up of 4 and 6 months, incidence of missed medication events in intervention group displayed depletion relative to that in controls. At follow-up of 4 and 6 months, patients’ medication compliance in intervention group displayed elevation relative to that in controls. These findings suggested that such management method can effectively reduce incidence of missed medication events and elevate patients’ medication compliance.

Herein, the incidence of adverse drug reactions displayed depletion in intervention group. At follow-up of 2, 4 and 6 months, incidence of erythra in intervention group displayed depletion relative to that in controls. Scholars have demonstrated that erythra is one of the most common adverse reactions of targeted drugs (19). Moreover, the incidence of erythra is related to the prognosis of NSCLC patients (20). The higher the incidence of erythra, the shorter the long-term survival period of patients. Adopting an oral targeted drug management program led by specialist nurses can elevate ability of patients, family members and primary caregivers to identify adverse drug reactions. In case of adverse reactions, responsible nurse should be contacted in a timely manner, and correct treatment methods should be taken under guidance of nurses to prevent serious adverse reactions. Herein, at follow-up of 6 months, general health, physical functioning and social functioning scores in intervention group displayed elevation relative to those in controls. The scores of above indicators in both groups at follow-up of 6 months displayed elevation relative to those at follow-up of 2 months. These findings suggested that adopting an oral targeted drug management program led by specialist nurses can effectively elevate patients’ quality of life.

In conclusion, an oral targeted drug management program led by specialist nurses can effectively elevate patients’ compliance, reduce incidence of adverse drug reactions and improve patients’ quality of life.

## Data Availability

The datasets presented in this study can be found in online repositories. The names of the repository/repositories and accession number(s) can be found in the article/supplementary material.
